# Monitoring Endothelin-A Receptor Expression during the Progression of Atherosclerosis

**DOI:** 10.3390/biomedicines8120538

**Published:** 2020-11-26

**Authors:** Miriam Stölting, Christiane Geyer, Anne Helfen, Anke Hahnenkamp, Marco V. Usai, Eva Wardelmann, Michael T. Kuhlmann, Moritz Wildgruber, Carsten Höltke

**Affiliations:** 1 Clinic for Radiology, University Hospital Münster, D-48149 Münster, Germany; Miriam.Stoelting@ukmuenster.de (M.S.); Christiane.Geyer@uni-muenster.de (C.G.); Anne.Helfen@ukmuenster.de (A.H.); Moritz.Wildgruber@ukmuenster.de (M.W.); 2 Department of Anesthesiology, University Medicine Greifswald, D-17475 Greifswald, Germany; Anke.Hahnenkamp@med.uni-greifswald.de; 3 Department of Vascular and Endovascular Surgery, University Hospital Münster, D-48149 Münster, Germany; MarcoVirgilio.Usai@sfh-muenster.de; 4 Gerhard-Domagk-Institute of Pathology, University Hospital Münster, D-48149 Münster, Germany; Eva.Wardelmann@ukmuenster.de; 5 European Institute for Molecular Imaging, Westphalian Wilhelms-University Münster, D-48149 Münster, Germany; Kuhlmam@uni-muenster.de; 6 Department of Radiology, University Hospital, LMU Munich, D-48149 Munich, Germany

**Keywords:** atherosclerosis, endothelin system, molecular imaging, ApoE-KnockOut, carotid endarterectomy, fluorescence imaging, endothelin receptor expression

## Abstract

Cardiovascular disease remains the most frequent cause of death worldwide. Atherosclerosis, an underlying cause of cardiovascular disease, is an inflammatory disorder associated with endothelial dysfunction. The endothelin system plays a crucial role in the pathogenesis of endothelial dysfunction and is involved in the development of atherosclerosis. We aimed to reveal the expression levels of the endothelin-A receptor (ET_A_R) in the course of atherogenesis to reveal possible time frames for targeted imaging and interventions. We used the ApoE^−/−^ mice model and human specimens and evaluated ET_A_R expression by quantitative rtPCR (qPCR), histology and fluorescence molecular imaging. We found a significant upregulation of ET_A_R after 22 weeks of high-fat diet in the aortae of ApoE^−/−^ mice. With regard to translation to human disease, we applied the fluorescent probe to fresh explants of human carotid and femoral artery specimens. The findings were correlated with qPCR and histology. While ET_A_R is upregulated during the progression of early atherosclerosis in the ApoE^−/−^ mouse model, we found that ET_A_R expression is substantially reduced in advanced human atherosclerotic plaques. Moreover, those expression changes were clearly depicted by fluorescence imaging using our in-house designed ET_A_R-Cy 5.5 probe confirming its specificity and potential use in future studies.

## 1. Introduction

Despite an improved prognosis for cardiovascular diseases, these remain the most frequent causes of death worldwide (WHO 2016). Most cardiovascular diseases, such as myocardial infarction or stroke, often occur due to rupture of atherosclerotic plaques, which are promoted by risk factors such as hyperlipidemia, hypertension, or diabetes. Nowadays, atherosclerosis is perceived as an inflammatory disease whose pathogenesis starts with endothelial dysfunction, which is associated with increased vascular permeability and the storage of cholesterol in the subintimal space. Subsequently, inflammatory cells are recruited into the vascular wall, resulting in a progressive growth and destabilization of the plaque. Vascular calcification and stenosis are believed to be only the final stages of a complex pathomechanism that can silently progress for a long time without clinical symptoms [1,2,3,4].

Endothelin-1 (ET-1) and its associated G-protein coupled receptors ET_A_R and ET_B_R are involved in the pathogenesis of atherosclerosis [5,6,7,8]. Additionally, this signaling axis also plays an important role in various tumor entities [9] and in the pathophysiology of pulmonary arterial hypertension (PAH), where ET receptor antagonism has shown clinical efficacy [10]. Under physiological conditions, the binding of ET-1 to ET_A_R, which is primarily expressed by vascular smooth muscle cells (VSMCs), leads to vasoconstriction. In contrast, ET_B_R is predominantly expressed by endothelial cells and is involved in ET-1 clearance and the initiation of NO synthesis, representing antagonistic actions concerning ET-1–ET_A_R signaling. The pathophysiological actions of ET-1 signaling in cardiovascular disease include effects on lipid uptake, stimulation of inflammation, VSMC-proliferation, and the formation of reactive oxygen species [6,11]. Changes of ET-1 and ET receptor expression have been detected in atherosclerotic mouse models and treating these mice with ET receptor antagonists lead to an attenuation of plaque formation [12,13]. In humans, changes in ET-1 and ET receptor expression as well as a reduction in plaque manifestation after endothelin receptor inhibition have also been shown. Winkles et al. found an increased ET-1 expression in atherosclerotic aortae, whereas ET receptors were reduced [14]. Moreover, long-term administration of the ET_A_R antagonist atrasentan improved coronary endothelial function in patients with endothelial dysfunction and reduced plaque formation [15]. Taken together there is strong evidence that ET receptors are crucial for atherogenesis and the understanding of their variable expression status in the course of plaque development is of tremendous importance for diagnostic and therapeutic approaches.

In diagnosis of atherosclerosis, vascular occlusions are primarily detected using classical imaging techniques such as CT-angiography, MRI or ultrasound. Although these methods typically allow the assessment of vascular or plaque anatomy, they are not suitable for the identification of molecular processes, biological activity within the lesion or plaque vulnerability [16,17]. Positron emission tomography (PET) imaging using ^18^F-fluorodeoxyglucose (FDG) mainly depicts the metabolism of plaque macrophages, which reflect local inflammatory processes. However, especially when it comes to coronary artery imaging, the myocardial background limits its use [18]. During the last years, innovative molecular optical imaging methods like fluorescence-mediated tomography (FMT), fluorescence-reflection imaging (FRI) or multispectral-optoacoustic tomography (MSOT) were established, which can be used with the aid of fluorescent probes for the early and specific in vivo detection of molecular processes in the preclinical setting [19,20,21,22,23]. Optical imaging is based on the identification of near-infrared (NIR) fluorescent substances within the organism and offers comparative sensitivity and spatial resolution as nuclear imaging methods. Fluorescent NIR dyes can be linked to target-specific structures (antagonists, antibodies) to build targeted contrast agents (probes) which can be visualized in vivo using the above-mentioned molecular imaging methods, thus paving the way for the delineation of certain cell populations, molecules and pathophysiological processes during the formation of atherosclerotic plaques.

The present study was based on the hypothesis that changes in the expression level of ET_A_R occur during atherogenesis and that these changes can be monitored by fluorescence molecular imaging, applying an ET_A_R specific small molecular probe. Therefore, we performed ET receptor expression analysis on a well-defined murine model of plaque formation (ApoE^−/−^ mice receiving a high-fat diet) as well as on human endarterectomy specimens from carotid and femoral arteries, where the presence of atherosclerotic plaques resulted in advanced manifestation of the disease.

## 2. Experimental Section

### 2.1. Chemistry and Reagents

All chemicals, reagents and solvents were analytical grade and purchased from commercial sources. The applied probe is based on a previously published small molecule fluorescent endothelin receptor antagonist. For the FRI approach, the fluorescent dye Cy 5.5 was used (ET_A_R-Cy 5.5), for additional MSOT experiments, IRDye800cw was coupled to the precursor compound (ET_A_R-IRDye) [24,25].

### 2.2. Histology and Immunofluorescence of Cryosections

After drying and pre-fixation in 4% paraformaldehyde (PFA, Santa Cruz Biotechnology, Dallas, TX, USA), cryosections were hematoxylin and eosin stained according to the manufacturer’s protocol (Morphisto; Frankfurt, Germany). Further cryosections were stained for ET_A_R (ab178454, ab76259, Abcam; Cambridge, UK), and α-smooth muscle actin (C6198, Sigma; St. Louis, MO, USA). Fluorophore-coupled secondary antibodies at 647 nm wavelengths (goat anti rabbit Alexa Fluor 647, Dianova 111-605-144; Dianova; Hamburg, Germany) were used for visualization. The mounting media Prolong Diamond (Invitrogen; Carlsbad, CA, USA) containing DAPI was used for tissue embedding and visualization of the cell nuclei. Negative control experiments were performed without first antibody. All slices were imaged using an Eclipse 50i microscope (Nikon; Tokio, Japan) and documented by NIS-Elements Br 3.22 software (Nikon).

### 2.3. Histology and Immunohistochemistry (IHC)

All tissue samples were fixated in 4% neutral buffered formalin for 24 h and subsequently embedded in paraffin according to standard protocols. Sections of 5 µm thickness were then manufactured using a microtome (RM2235, Leica; Wetzlar, Germany). Sections were stained for ET_A_R (ab178454, ab76259, Abcam) using a Vectastain kit (PK-6101, Vector Labs; Burlingame, CA, USA) with peroxidase substrate (SK-4100, Vector Labs). Further stainings included hematoxylin and eosin (Morphisto), Mac-3 (BD550292, BD Biosciences; Heidelberg, Germany) and Elastica van Gieson (Morphisto), applied according to the manufacturer’s protocol. Negative control experiments were performed without first antibody. All sections were scanned using an Eclipse 50i microscope (Nikon) and documented using NIS-Elements Br 3.22 software (Nikon).

### 2.4. Patient Recruitment and Tissue Extraction

Patients (mean age vein stripping patients 40 years, endarterectomy patients 70 years; both genders) scheduled for endarterectomy or vein stripping at the Department of Vascular and Endovascular Surgery at the University Hospital Münster were recruited for the study after informed consent had been obtained. The human study was approved by the ethical committee of the Westfälische Wilhelms-Universität Münster (protocol number 2016-419-f-S). Preoperative evaluation of the patients was performed according to the local protocols. Endarterectomy of carotid or femoral plaques was performed under general anesthesia using standard techniques [26]. All endarterectomy patients received intravenous heparin bolus dose (5000 I.U.) during the procedure. The explanted plaques were directly transferred into Eppendorf tubes filled with 1.0 µM ice-cold ET_A_R-Cy 5.5 in PBS. During crossectomy of the vena saphena magna using a standard procedure [27], a small part of the external pudendal artery was extracted and similarly transferred into an Eppendorf tube containing 1.0 µM ice-cold ET_A_R-Cy 5.5 in PBS. All explanted tissues were incubated with the probe for 30 min at 0 °C. Afterwards, specimens were washed twice with PBS, cut in two to four preferably similarly sized pieces (in case of carotid or femoral plaques), dried and weighed before fluorescence reflectance imaging was performed.

### 2.5. Mice

Animal experiments were performed in accordance with the national and European legislation for animal care and experiments and were approved by the animal ethics committee of the Landesamt für Natur, Umwelt und Verbraucherschutz Northrhine Wesfalia (regional authority for animal ethics LANUV NRW; License numbers: 84-02.04.2016.A511). Female apolipoprotein E deficient (ApoE^−/−^) mice at the age of 8–12 weeks were obtained from Charles River Laboratories (Sulzfeld, Germany), wild-type C57Bl/6 mice were received from the animal facility at the University Hospital Münster. Mice received a high-fat diet containing 0.15% cholesterol and 21% total fat (Altromin Western-Type diet, Altromin Spezialfutter GmbH & Co. KG, Lage, Germany) for 6, 12 or 22 weeks or a standard diet without cholesterol and 4% total fat (Altromin 1324, Altromin Spezialfutter GmbH & Co. KG). For Imaging, mice received 2.0 nmol of the fluorescent probe dissolved in physiological saline solution via tail vein injection. After 24 h the aortic arch was excised and transferred to near infrared imaging. For excision of the aortic arch mice were euthanized under deep anesthesia using isofluorane (1.5–2.5% *v*/*v* in O_2_).

### 2.6. Imaging

Near infrared FRI was performed using the In-Vivo FX Pro Imaging System (Bruker BioSpin GmbH, Rheinstetten, Germany) equipped with a 400-W halogen illuminator with Cy 5.5 bandpass excitation (625 ± 18.0 nm) and emission filters (700 ± 17.5 nm). Fluorescence signals were captured with a 4-million-pixel cooled charge-coupled device (CCD) camera equipped with a 10X zoom lens. Images were captured 24 h after probe injection, with an acquisition time of 5 s and identical window settings (binning, f-stop, field of view). Excised mice aortae or human specimen were placed on a Petri dish for fluorescence imaging and signal intensity was recorded. Fluorescence images were analyzed and co-registered with the anatomic white light images using the Bruker MI 7.5 software (Bruker BioSpin GmbH).

### 2.7. Tissue Preparation and RNA Isolation

After NIR imaging, mice and human tissues were either embedded for histology in 4% formalin (paraffine sections) or TissueTEK O.C.T. (cryosections) or shock frozen in liquid nitrogen for isolation of total RNA. Prior RNA isolation the frozen tissues were homogenized in TRIZOL using the Precellys Evolution homogenizer (Bertin Technologies SAS, Montigny-le-Bretonneux, France) by a user-defined program. RNA isolation was performed with a Qiagen RNeasy Kit (Qiagen, Hilden, Germany) according to the manufacturer’s instructions. RNA quality was monitored using the Bioanalyzer 2100 (Agilent, St. Clara, CA, USA) of the Core facility Genomics at the University of Münster. Probes with an RNA integrity value above 5 were chosen for further qPCR analysis.

### 2.8. qPCR

Quantitative real-time PCR was performed using the Eppendorf Real Plex Cycler (Eppendorf; Hamburg, Germany) with the KAPA Sybr Fast OneStep qPCR Kit (Sigma) and 20 pg of total RNA according to the manufacture’s protocol and with Quantitect primer assays (Qiagen, Hilden, Germany; primer details see Table S1, Supplementary Material). Glyceraldehyde 3-phosphate dehydrogenase (GAPDH) and peptidyl-prolyl cis-trans isomerase B (PPiB) were used as house-keeping (reference) genes. Examples of PCR products were analyzed by agarose gel electrophoresis. Analyses of qPCR data were performed using the Relative Expression Software Tool (REST, Qiagen, see below). Data were processed by Microsoft Excel and visualized with GraphPad Prism 7.02.

### 2.9. Statistical Analysis

Data are presented as mean ± standard error (SEM) or as box and min-to-max whiskers. qPCR data were analyzed using the REST software, which uses a fixed reallocation randomization test for determination of significant differences [28], and further processed and visualized with GraphPad Prism 7.02. FRI data were processed with GraphPad Prism 7.02. One-way ANOVA analysis with Dunnett’s post-test was used for intra-strain analysis, while a two-way ANOVA and Tukey’s post-test was used for the inter-strain comparison. A *p*-value < 0.05 was considered statistically significant.

## 3. Results

### 3.1. ApoE^−/−^ Mice Show Enhanced ET_A_R Expression after High-Fat Diet

**Figure 1:**
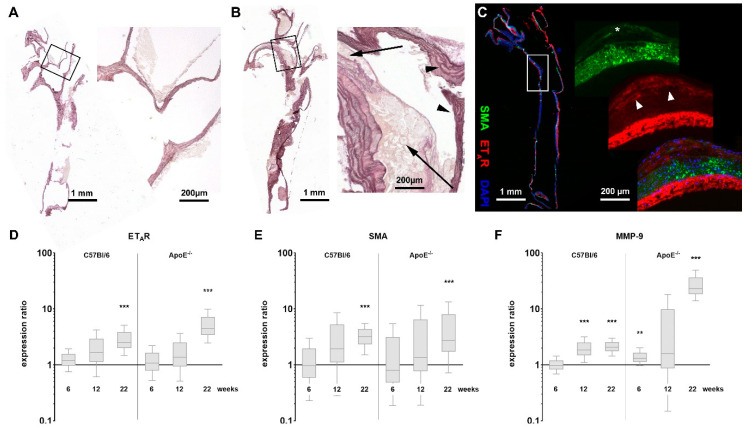
Histology and qPCR results indicate upregulation of ET_A_R in atherosclerotic tissue of ApoE^−/−^ mice. (**A**) Elastica van Gieson (EvG) staining of an explanted healthy aortic arch of a control C57Bl/6 mouse without high-fat diet. The magnification showing the bifurcation of the left carotid artery. (**B**) EvG staining of an aorta from an ApoE^−/−^ mouse after 22 weeks of high-fat diet showing extensive plaque deposits. The magnification shows both affected (arrows) and unaffected vessel walls (arrowheads) around the bifurcation. (**C**) Immunofluorescence staining, depicting ET_A_R (red) and SMA (green) expression in a vessel from an ApoE^−/−^ mouse after 12 weeks of high-fat diet (+ DAPI nuclear stain, blue). A developing plaque can be localized at the left vessel wall. Inside the plaque SMA (asterisk) and ET_A_R (arrowheads) from proliferating smooth muscle cells are co-resident. The prominent signal of ET_A_R within the adventitia is possibly in part due to adventitial fibroblasts or macrophages infiltrating the diseased vascular wall. (**D**–**F**) qPCR comparison of ET_A_R, SMA and MMP-9 expression in aortic tissue of ApoE^−/−^ mice (*n* = 6–11) and C57Bl/6 mice (*n* = 3–11) after indicated time of high-fat diet vs. *t* = 0 (asterisks indicate significance: ** *p* < 0.01, *** *p* < 0.005).

In this study, we investigated the expression of the endothelin-A receptor (ET_A_R) in ApoE^−/−^ animals fed a high-fat diet for 6, 12 or 22 weeks and compared the results with wild-type C57Bl/6 mice receiving a high-fat diet as well as to animals without high-fat diet. Elastica van Gieson (EvG) staining of mouse aortae revealed structural changes of the aortic vessels during diet-induced plaque development. While the aorta of a C57Bl/6 mouse prior to a high-fat diet shows no atherosclerotic changes in the vessel wall (Figure 1A), long-term high-fat diet for 22 weeks reveals a high load of large fat-rich plaques around the bifurcation in the aortic arch of the ApoE^−/−^ mouse aorta (Figure 1B) as typically observed during atherosclerosis development in humans. Additionally, the immunostainings for ET_A_R and SMA in an aorta from an ApoE^−/−^ mouse after 12 weeks of high-fat diet presented in Figure 1C, show an emerging plaque structure within the vessel wall indicating the onset of vascular remodeling (control image w/o first antibody see Figure S1A). The presence of SMA as well as ET_A_R within the plaque suggests the proliferation of smooth muscle cells into the early atherosclerotic lesion. Aortae from C57Bl/6 mice after 12 weeks of high-fat diet do not show any signs of plaque development or high ET_A_R expression (Figure S1B). The prominent ET_A_R signal from the adventitia in Figure 1C might at least in part be due to the presence of adventitial fibroblasts, which are known to contribute to increased levels of collagen type I expression associated with fibrosis, increased vessel stiffness and VSMC migration by ET-1 signaling [29]. Mac-3 staining for macrophages shows an enhanced presence of immune cells within the plaque wall (Figure S1C), which might also be a source of ET_A_R probe fluorescence [30]. Analysis of ET_A_R, SMA and MMP-9 mRNA expression during the diet-induced progression of atherosclerosis reveals marked differences between ApoE^−/−^ and control animals (Figure 1D–F). Quantitative qPCR analyses of ET_A_R expression show a significant upregulation in ApoE^−/−^ mice aortae as well as in aortae of wild-type mice (Figure 1D), but the enhancement was > 4-fold in ApoE^−/−^ mice and twofold in control mice. After 6 and 12 weeks of diet, no significant differences in ET_A_R expression were detected. Expression of SMA seems to be similarly regulated in both strains (Figure 1E). A significant two- to threefold upregulation can be observed after 22 weeks of diet. The expression of MMP-9, which serves as an established marker for inflammation during atherosclerosis progression, shows a strong upregulation in ApoE^−/−^ mice due to high-fat diet induced plaque development after 22 weeks (Figure 1F), while control animals only show a low upregulation. An inter-strain comparison of the most severe atherosclerotic state (22 weeks of diet in ApoE^−/−^ mice) with putatively healthy aortae from C57Bl/6 mice without high-fat diet indicates a significant upregulation of all evaluated genes with MMP-9 being the most prominent and SMA being the least affected target (Figure S2A). A comparison to age- and diet-matched wild-type mice also shows upregulated genes for MMP-9 and ET_A_R, but SMA seems downregulated (Figure S2B).

### 3.2. Molecular Imaging Shows Increased Probe Uptake in Aortic Lesions of ApoE^−/−^ Mice

**Figure 2:**
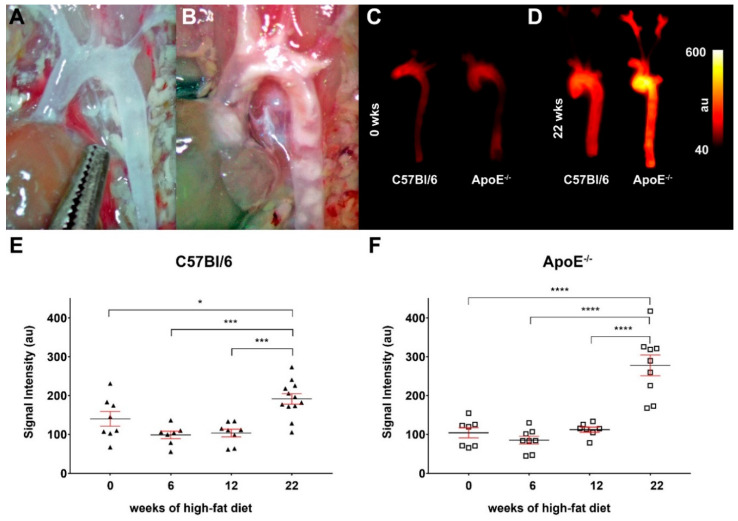
Fluorescence imaging of ApoE^−/−^ aortae with the ET_A_R-targeted probe show enhanced signal intensity after 22 weeks of high-fat diet. (**A**,**B**) Images of aortic arches of a C57Bl6 mice (**A**) and an ApoE^−/−^ mouse (**B**) after 22 weeks of high-fat diet prior to extraction. A high amount of plaque lesions/calcifications can be observed in the aortae of ApoE^−/−^ mice. (**C**,**D**) Fluorescence reflectance images of aortic arches of C57Bl6 (left) and ApoE^−/−^ mice (right) before (0 weeks, (**C**)) and after 22 weeks of high-fat diet (**D**). Images were captured directly after extraction of the aortic arch, 24 h after injection of 2.0 nmol of the probe. (**E**,**F**) Graphs showing the detected fluorescence intensities (means ± SEM, all data points indicated) of ex vivo imaged aortae of C57Bl/6 (triangles, (**E**)) and ApoE^−/−^ mice (squares, (**F**)) before (0 weeks, *n* = 8 vs. *n* = 8) and after 6 weeks (*n* = 7 vs. *n* = 8), 12 weeks (*n* = 8 vs. *n* = 7) and 22 weeks (*n* = 12 vs. *n* = 9) of high-fat diet. Significant differences could be identified by one-way ANOVA after the longest period of diet in both strains (* *p* < 0.05, *** *p* < 0.005, **** *p* < 0.001).

Using a small molecular fluorescent probe consisting of an ET_A_R ligand, a short spacer and Cy 5.5 as fluorescent dye, we were able to visualize alterations in ET_A_R expression by means of fluorescence reflectance imaging. Figure 2A,B show aortae of a control and an ApoE^−/−^ animals after 22 weeks on high-fat diet prior excision and imaging. Atherosclerotic plaques can be perceived as white blotches within the ApoE^−/−^ aorta. Ex vivo FRI of aortae before high-fat diet depict similar low mean fluorescence intensities in the control and ApoE^−/−^ vessel (Figure 2C). Mean fluorescence intensities are increased in aortae of both mice types after 22 weeks of high-fat diet, with the aortae of ApoE^−/−^ mice showing significantly higher intensity values (Figure 2D). Analyses of mean fluorescence intensities in aortae of control and ApoE^−/−^ mice after 0, 6, 12 and 22 weeks of high-fat diet demonstrate that within the first weeks of diet only slight differences between the two strains are visible in a range of 70 to 150 absorption units (au). Figure 2E,F show that after 22 weeks of diet, the mean fluorescence intensities of control and ApoE^−/−^ aortae are significantly elevated. ApoE^−/−^ tissue shows higher intensity values than tissue from C57Bl/6 mice. An inter-strain analysis also reveals significantly higher values of ApoE^−/−^ aortae after 22 weeks of diet compared to the control aortae after the same diet and compared to putatively healthy tissue from wild-type mice without diet (Figure S2C). These findings point to a stronger ET_A_R expression in ApoE^−/−^ aortae during atherosclerotic plaque development after 22 weeks of cholesterol-rich diet, but also to a marked increase in wild-type animals.

### 3.3. Expression of ET_A_R Is Reduced in Advanced Human Endarterectomy Specimens

**Figure 3:**
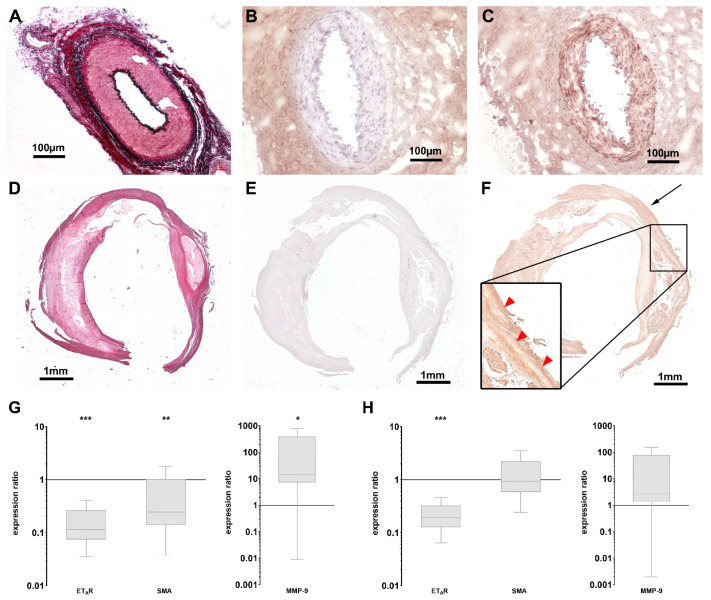
Expression of ET_A_R in human atherosclerotic plaques is downregulated. (**A**–**C**) Histology of a human artery pudenda externa as healthy control stained with EvG (**A**) and for ET_A_R (**C**) showing a strong ET_A_R expression within the media ((**B**) negative control w/o first antibody). (**D**–**F**) Histology of paraffin-embedded human carotid artery specimen after endarterectomy stained with EvG (**D**) and for ET_A_R (**F**) showing advanced atherosclerotic lesions with locally reduced staining for ET_A_R within the lesion (red arrowheads in magnification), compared to putatively healthy tissue (black arrow. (**E**) negative control w/o first antibody). (**G**,**H**) Box plots with min-to-max whiskers of qPCR data showing the expression ratios of ET_A_R, SMA (left) and MMP-9 (right) in atherosclerotic carotid specimen ((**G**) *n* = 8) versus healthy arteries (*n* = 7) and atherosclerotic femoral specimen ((**H**) *n* = 8) versus healthy arteries (*n* = 7). Significant reductions in ET_A_R expression were found in both lesions, while SMA is only significantly reduced in carotid tissue. MMP-9 was significantly elevated in specimen from carotid arteries, but not from femoral arteries (* *p* < 0.05; ** *p* < 0.01; *** *p* < 0.005).

To explore possible translational aspects of these findings, we examined specimen of human carotid and femoral explants with advanced plaques after endarterectomy concerning their ET_A_R expression profiles. Parts of the external pudendal artery, which were removed during routine varicose vein surgery, served as healthy control. Incubation of the excised tissue specimen with 1.0 µM ET_A_R-Cy 5.5 immediately after surgery and subsequent fluorescence reflectance imaging was followed by histology and qPCR examinations. EvG staining of healthy arteries showed intact vascular smooth muscle layers and surrounding elastic fibers within the vessel structures (Figure 3A), control image w/o first antibody see Figure 3B. The expression of ET_A_R was uniformly distributed within the smooth muscle layer (Figure 3C). Atherosclerotic specimens showed a less uniform EvG staining (Figure 3D), control image w/o first antibody see Figure 3E and also ET_A_R distribution was heterogeneously distributed in regions of atherosclerotic transformations (Figure 3F). qPCR investigations proved these findings and showed an overall reduced degree of ET_A_R and SMA expression within carotid explants compared to healthy arteries, while MMP-9 was upregulated (Figure 3G). In femoral explants, a reduction in ET_A_R expression could also be proven, as well as an upregulation of MMP-9. SMA did not show significant variations (Figure 3H).

### 3.4. Reduced ET_A_R Expression in Human Atherosclerotic Plaques Can Be Depicted by FRI

**Figure 4:**
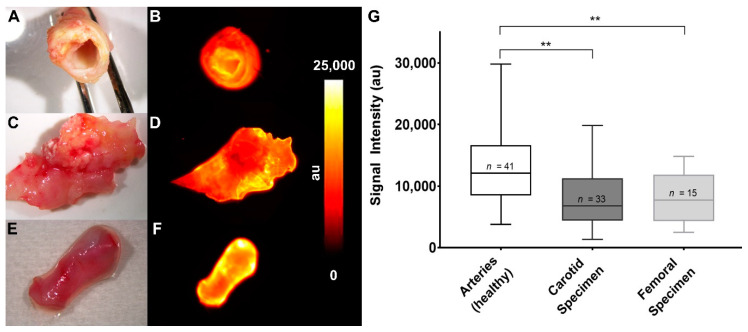
Fluorescent imaging of human specimen shows reduced signal intensities in atherosclerotic carotid and femoral tissue. (**A**,**B**) Human carotid specimen after incubation in 1.0 µM ET_A_R-Cy 5.5 for 30 min. (**A**) Color photograph. (**B**) Fluorescent image. (**C**,**D**) Human femoral tissue after extraction and incubation. (**C**) Color photograph. (**D**) Fluorescent image. (**E**,**F**) Piece of the arteria pudenda externa. (**E**) Color photograph. (**F**) Fluorescent image. (**G**) Graphical analysis of fluorescence intensities from human specimen after incubation in 1.0 µM ET_A_R-Cy 5.5 for 30 min, indicating a significant reduction of ET_A_R expression in atherosclerotic tissue (** *p* < 0.01).

Fluorescence reflectance imaging of the explants immediately after surgery showed a reduced accumulation of the ET_A_R-Cy 5.5 probe in endarterectomy specimen compared to arteries. Figure 4 shows color photographs (Figure 4A,C,E) and fluorescence images (Figure 4B,D,F) of all three explanted tissue types. Whereas healthy arteries show a homogenous fluorescence (Figure 4B) fluorescent signal distribution was generally rather heterogeneous in the carotid and femoral specimen. Nevertheless, analysis of signal intensities of carotid and femoral explants in comparison to healthy specimen showed a significantly reduced mean fluorescence intensity (Figure 4G). To prove that the ET_A_R probe can be useful for additional molecular imaging techniques, we employed ET_A_R-IRDye as probe and examined a femoral artery specimen in an agarose gel composition by Multispectral Optoacoustic Tomography (MSOT) [19,31]. This technique combines the use of molecular fluorescence imaging with an ultrasound readout. Using MSOT, probe distribution was clearly delineated within the specimen inside the agarose gel with high spatial resolution (Figure S3).

## 4. Discussion

In the present study, we investigated the time course of ET_A_R expression in high-fat diet-induced aortic atherosclerosis in ApoE^−/−^ mice and in human advanced atherosclerotic plaques in correlation to smooth muscle actin (SMA) and matrix metalloproteinase 9 (MMP-9). Moreover, we monitored the expression changes of ET_A_R not only by molecular biology methods but by molecular imaging using an ET_A_R-specific fluorescent probe. For exploration in mice, we chose the homozygous apolipoprotein E-deficient mouse strain (ApoE^−/−^), which has long been used as a model for atherogenesis. ApoE plays a central role in lipoprotein metabolism and is required for the clearance of diet-derived chylomicrons and liver-derived vLDL by the liver [32]. Consequently, mice lacking ApoE can be used as an animal model of hyperlipidemia (especially hypercholesterolemia) and resulting atherosclerosis, which can be accelerated by feeding a high-fat (western-type) diet [33].

Here, we were able to detect significant differences in ApoE^−/−^ mice after 22 weeks of high-fat diet, where qPCR as well as FRI data showed an upregulation of ET_A_R expression. C57Bl/6 mice also show an ET_A_R upregulation due to the high-fat diet, but this is independent of atherosclerotic plaque development and lower than in the ApoE^−/−^ mice. Therefore, we suggest that the observed upregulation in ApoE^−/−^ mice reflects the effect of developing atherosclerosis. These results are in line with further studies that investigated the ET system in atherosclerotic ApoE^−/−^ mice. Barton and colleagues described an enhanced expression of ET-1 as well as ET_A_R in ApoE^−/−^ mice after 30 weeks of high-fat diet, which they discovered by protein extraction and autoradiography, respectively. Feeding an ET_A_R antagonist was able to prevent exacerbation of high-fat diet induced atherosclerosis, including reduction of tissue ET-1 levels [13]. Maguire et al. in 2006 showed that ET-1 mediated vasoconstriction in ApoE^−/−^ mice is enhanced compared to C57Bl/6 mice, indicating a contribution of the endothelin system to disease progression [34]. They also investigated ET receptor expression and found no differences in young normally fed ApoE^−/−^ mice compared to C57Bl/6 controls. Additionally, it has been described that the vascular structure and function of mesenteric arteries of ApoE^−/−^ mice fed a normal chow diet did not differ from C57Bl/6 mice fed a western-type diet but were significantly altered in western-type diet fed ApoE^−/−^ mice [35]. Adverse effects were mostly reversible by ET_A_R antagonism. In a porcine model of hypercholesterolemia, the authors described that coronary vasa vasorum neovascularization occurs within the first weeks of high-fat diet [36]. Since ET-1 is a known mediator of angiogenesis, it is conceivable, that these processes are resulting in altered ET receptor expression within affected tissue [37,38,39,40]. In a study by Kobayashi et al. in ApoE^−/−^ mice, increased immunostaining of ET_A_R—specifically in medial VSMCs—is described [41]. α-Smooth muscle actin (SMA) is a marker mainly expressed by VSMCs. We chose SMA for the correlation of VSMC-derived ET_A_R within healthy and diseased tissue. Already after 12 weeks of high-fat diet we identified SMA in the fibrous cap of developing aortic plaques, presumably due to proliferating VSMCs, also expressing ET_A_R (Figure 1C). In addition, macrophages were depicted by Mac-3 staining within the plaque border (Figure S1C). Macrophages express components of the endothelin system including ET_A_R, but are also capable of inducing ET-1 secretion and ET receptor expression in vitro in a variety of cell types including HUVECs [30,42,43]. Moreover, neutrophils also express ET_A_R [43], so the observed, SMA-independent ET_A_R staining might arise from these inflammatory cells We showed that qPCR analysis of SMA gene expression confirms the histological observation as it shows an increase after 22 weeks of high-fat diet. During atherogenesis a switch of VSMCs towards a more macrophage-like phenotype is observed, showing reduced expression of SMA [44,45]. This may explain the merely moderate rise in SMA compared to ET_A_R and the reduced expression of SMA within ApoE^−/−^ aortae compared to C57Bl/6 tissue.

The fact that immunohistochemistry showed alterations within aortic vascular structures as early as 12 weeks after diet onset (Figure 1), where emerging plaque structures could be identified, was supported by a study from Shon et al., which showed plaque development as early as 8 weeks after onset of a high-fat diet in ApoE^−/−^ mice [46]. Ex vivo FRI using an ET_A_R-specific, Cy 5.5-coupled antagonist reflects the described expression patterns, showing a stronger mean fluorescence intensity in atherosclerotic aortae of ApoE^−/−^ mice due to increased ET_A_R expression.

As a consequence, we decided to monitor ET_A_R expression in explants of human atherosclerotic plaques and healthy arteries not only by immunohistochemistry and qPCR but also by ex vivo FRI. Interestingly, in human endarterectomies of advanced atherosclerotic plaques versus healthy arteries we observed an opposing trend compared to mouse data. This might be explained by the fact that high-fat induced atherosclerosis in ApoE^−/−^ mice mostly represents a hyperlipidemia-accelerated process when compared to human atherosclerotic conditions [47]. Therefore, it only in part reflects the heterogeneity of the human specimen that were used in this study. Nevertheless, ApoE^−/−^ mice develop an entire spectrum of lesions similar to human atherosclerotic plaques including foam cell lesion after 8 weeks of diet, early fibrous lesions after 15 weeks of diet and more advanced stages, characterized by a higher number of proliferating VSMCs and monocytes and calcifications after 60 weeks of diet [47]. The observed upregulation of ET_A_R in ApoE^−/−^ mice after 22 weeks of diet is therefore an attribute of early plaque development, while the ET_A_R downregulation in human specimen is attributable to advanced atherosclerotic plaque physiology.

The observed differential findings are supported by a recent study of Rafnsson et al. They investigated the expression profiles of endothelin system components in carotid plaques from human endarterectomies from the Biobank of Karolinska Endarterectomies (BiKE) [48]. While ET-1 and ET_B_R were upregulated, Rafnsson et al. also found a downregulation of ET_A_R. Nevertheless, the inclusion of femoral plaques in our study, which show similar expression profiles, is an add-on to their results as it indicates that the observed ET_A_R downregulation might be a common outcome of atherosclerotic plaque development also in peripheral arteries. Moreover, studies have shown that femoral plaques may serve as a better indicator for cardiovascular risk as they show stronger association with cardiovascular risk factors and coronary calcium score [49].

The obtained results on the kinetic course of ET_A_R expression allowed to address different expressions by means of molecular imaging, which could ultimately help to predict the vulnerability of atherosclerotic plaques in vivo [50]. However, its clinical translation is still limited; on the one hand due to the lack of applicable contrast agents, and on the other hand, due to the lack of approved techniques. In particular, optical imaging of (cardio) vascular disease in vivo is challenging since deep-lying vessels are hardly to depict through the overlying tissue. This holds true not only for clinical applications, but also for preclinical animal models like ApoE^−/−^ mice. Thus, for a (semi) quantitative assessment of plaque components inside the aortic vessels, at the moment optical imaging is still restricted to ex vivo evaluation of fluoroprobe distribution [51,52,53,54]. In this setting, our probe is capable of delineating regions of higher intensity within the aortic arch, summing up to an overall higher signal intensity of diseased vessels versus control and also depicts the lower amount of ET_A_R in human atherosclerotic plaques. Moreover, it can be used as a tool to add a distinct imaging signature to quantitative techniques like qPCR. As a first pilot experiment, we went one step further and performed a proof of principle examination using ET_A_R-IRDye probe in combination with the emerging MSOT technology. We observed a clear delineation of the ET_A_R-IRDye-enriched areas within the femoral plaque (Figure S3). As this technique is already in clinical translation it could be a powerful, standalone tool for future molecular imaging approaches, especially when combined with suitable fluorescent probes [55,56].

Limitations of our study include the lack of healthy carotid and femoral specimens from human origin, so that a comparison within the same vessel type was not possible. However, we think that in light of the histology results the external pudendal artery can serve as a reasonable healthy control in this setting. Rafnsson et al. used macroscopically disease-free iliac arteries and one aorta from organ donors without a history of cardiovascular disease as control tissue. An evaluation of modifications in cellular and matrix structure in peripheral vessels disclosed that variations mainly occur with aging and with occurrence of vascular disease, e.g., atherosclerosis. However, these changes occur more or less similarly in all peripheral vessels [57]. In general, the direct comparison of artificial murine atherosclerosis to human endarterectomy specimen proved difficult as the extracted specimen contain highly advanced stages of atherosclerotic disease and are highly heterogenous concerning their origin, which is not reflected by available murine models. The lack of age-matched controls in the animal experiments might lead to a diverse interpretation of resulting data, especially concerning the degree of target upregulation. However, the purpose of our study was to investigate the effect of atherosclerosis on target expression, not the inherent effect of the high-fat diet, as this was shown before [13,34,35]. In light of the large amount of available data from the literature, which confirms our findings regarding the change of expression profile of ET_A_R in the examined tissue, we think that our conclusions are eligible.

## 5. Conclusions

Our study presents the course of ET_A_R expression in early atherosclerosis of ApoE^−/−^ mice and in advanced atherosclerotic plaques from humans by combining quantitative molecular biology as well as molecular imaging techniques. The applied fluorescent ET_A_R probe as a molecular imaging tool accurately depicts receptor distribution within murine and human atherosclerotic tissue in different disease stages. Interestingly, it reveals a different receptor expression time course in the two models, emphasizing the relevance of ET signaling in atherogenesis. Our findings support a further use of this probe in molecular imaging approaches targeting ET_A_R. Additionally, we show that the murine ApoE^−/−^ model might not be an ideal template for the comparison to human plaque physiology. A more heterogenous model might be more suitable.

## Supplementary Materials

The following are available online at https://www.mdpi.com/2227-9059/8/12/538/s1.
