# Supplementary material for: Monitoring Endothelin-A Receptor Expression during the Progression of Atherosclerosis
Source: Biomedicines. 2020 Nov 26;8(12):538. doi: 10.3390/biomedicines8120538 (PMC7761144; doi:10.3390/biomedicines8120538)
Supplement: Supplementary file 1 [file biomedicines-08-00538-s001.pdf]

Monitoring Endothelin A Receptor Expression during the Progression of Atherosclerosis

Miriam Stölting[1], Christiane Geyer[1], Anne Helfen[1], Anke Hahnenkamp[2], Marco V. Usai[3], Eva Wardelmann[4], Michael T. Kuhlmann[5], Moritz Wildgruber[1,6] and Carsten Höltke[1,*]

1) Clinic for Radiology, University Hospital Münster, Münster, Germany; 2) Department of Anesthesiology, University Medicine Greifswald, Greifswald, Germany; 3) Department of Vascular and Endovascular Surgery, University Hospital Münster, Münster, Germany; 4) Gerhard-Domagk-Institute of Pathology, University Hospital Münster, Münster, Germany; 5) European Institute for Molecular Imaging, Westfälische Wilhelms-Universität Münster, Münster, Germany; 6) Department of Radiology, University Hospital, LMU Munich, Munich, Germany.

Supplementary Information

Table 1. Primer information for qPCR analysis.

Target	Human	Order-no.	Mouse	Order-no.
GAPDH	Hs_GAPDH_1_SG	QT00079247	Mm_Gapdh_3_SG	QT01658692
PPiB	Hs_PPIB_1_SG	QT00067186	Mm_Ppib_1_SG	QT00169736
ET_AR	Hs_EDNRA_1_SG	QT00030156	Mm_Ednra_1_SG	QT00121625
SMA	Hs_ACTA2_1_SG	QT00088102	Mm_Acta2_1_SG	QT00140119
MMP-9	Hs_MMP9_1_SG	QT00040040	Mm_Mmp9_1_SG	QT01658692

Figure S1. Additional immunohistochemical images.

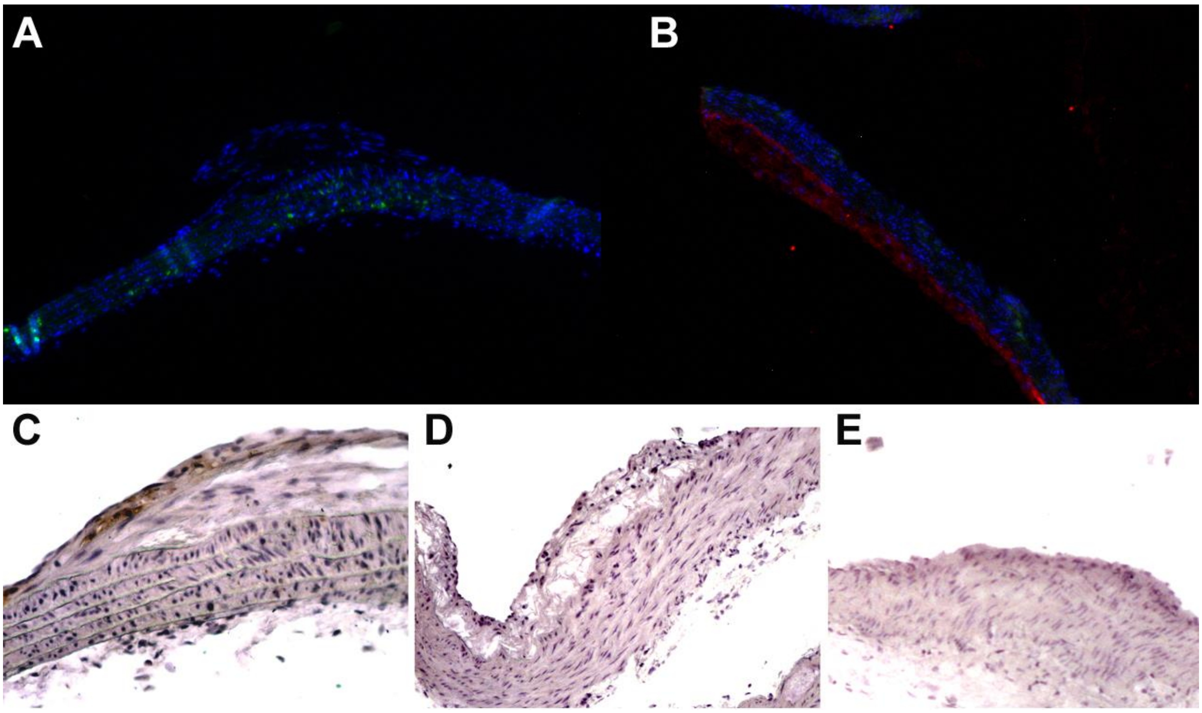

Figure S1. Additional histological images. Negative controls and analysis of macrophages. A. Negative control image corresponding to figure **1C** showing the same plaque structure without addition of the first ET_AR antibody. **B.** Control experiment corresponding to images in figure **1C** showing parts of

an aorta of a C57Bl/6 mouse after 12 weeks of high-fat diet showing no sign of enhanced ET$_A$R expression. **C.** An emerging plaque from an aorta of an ApoE$^{-/-}$ mouse fed a high-fat diet for 12 weeks already shows enhanced presence of Mac-3-positive cells in the fibrous cap (brownish colour, **D.** negative control without first antibody). **E.** The arterial wall of a C57Bl/6 control animal does not show enhanced staining for Mac-3 after 12 weeks of high-fat diet.

Figure S2. Inter-strain comparison of target gene expression and aorta fluorescence intensity.

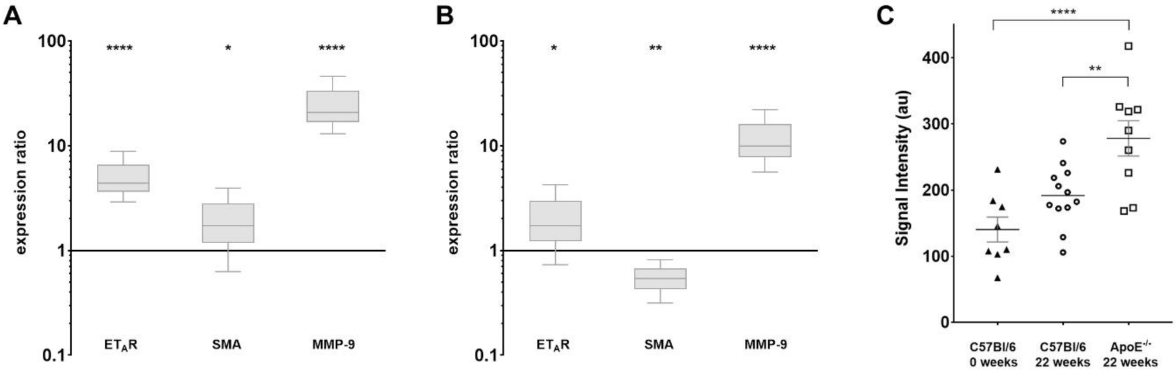

Figure S2. **Inter-strain comparison of target gene expression and aorta fluorescence intensity.** **A.** Analysis of target gene expression in ApoE$^{-/-}$ aorta after 22 weeks of high-fat diet compared to putatively healthy aorta of normally fed C57Bl/6 animals. **B.** Analysis of target gene expression in ApoE$^{-/-}$ aorta after 22 weeks of high-fat diet compared to aorta of age-matched C57Bl/6 animals fed the same diet. **C.** Comparison of fluorescence intensities from aortae of ApoE$^{-/-}$ animals after 22 weeks of high-fat diet (n = 9), aortae of putatively healthy, normally fed C57Bl/6 animals (0 weeks, n = 8) and of age-matched C57Bl/6 animals fed the same diet (22 weeks, n = 12). Two-way ANOVA analysis reveals significant differences. Asterisks indicate significant differences (* $p < 0.05$; ** $p < 0.01$; **** $p < 0.001$).

Figure S3. MSOT examination of a femoral sample after endarterectomy.

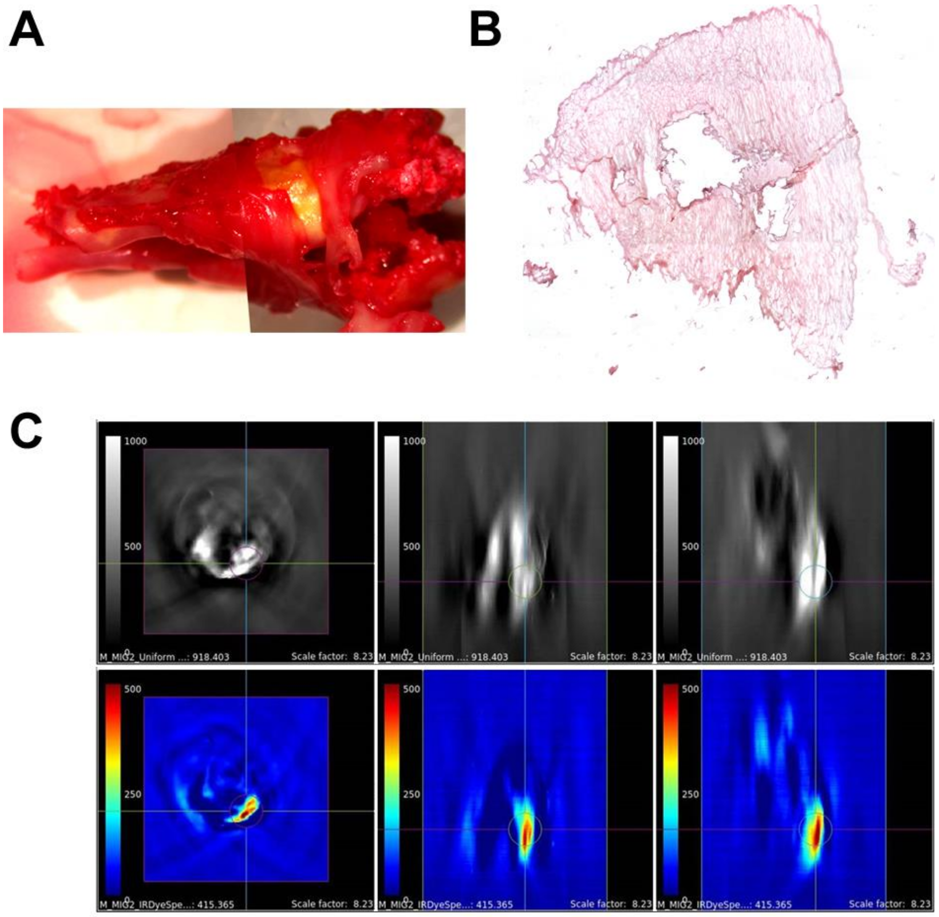

Figure S3. **Exemplary visualization of MSOT imaging results from a femoral plaque specimen. A**. Colour photography of the sample (composed image). **B**. Elastica van Gieson staining of a cross section of the sample. **C**. MSOT images of the agarose embedded sample showing the geometry of the sample (upper row, uniform absorber spectrum) and the distribution of the fluorescent probe ET$_A$R-IRDye (bottom row, IRDye800cw spectrum) in different orientations (transversal, frontal, sagittal, from left to right). Excitation wavelengths were 684, 700, 715, 730, 744, 760, 773, 800 and 850nm, with 10 frames each. The field of view was 25mm^3 at a resolution of 75µm. Image reconstruction was performed via a software embedded backprojection method, spectral unmixing was then utilized to resolve signals corresponding to individual chromophores using least-squares linear regression.
